# Expression of Stemness Markers in the Cervical Smear of Patients with Cervical Insufficiency

**DOI:** 10.3390/cells12081183

**Published:** 2023-04-18

**Authors:** Savvia Pittokopitou, Despina Mavrogianni, Vasilios Pergialiotis, Kalliopi I. Pappa, Panagiotis Antsaklis, Marianna Theodora, Michail Sindos, Angeliki Papapanagiotou, Aikaterini Domali, Sofoklis Stavros, Peter Drakakis, George Daskalakis

**Affiliations:** 1 1st Department of Obstetrics and Gynecology, Division of Gynecologic Oncology, Alexandra Hospital, National and Kapodistrian University of Athens, 11528 Athens, Greece; 2 Department of Biological Chemistry, Medical School, National and Kapodistrial University of Athens, 11528 Athens, Greece

**Keywords:** preterm birth, cervical insufficiency, OCT-4, DAZL, Nanog, stemness markers

## Abstract

The presence of stem cells has been previously described in human precancerous and malignant cervical cultures. Previous studies have shown a direct interplay of the stem cell niche, which is present in practically every tissue with the extracellular matrix. In the present study, we sought to determine the expression of stemness markers in cytological specimens collected from the ectocervix among women with cervical insufficiency during the second trimester of pregnancy and women with normal cervical length. A prospective cohort of 59 women was enrolled of whom 41 were diagnosed with cervical insufficiency. The expression of OCT-4 and NANOG was higher in the cervical insufficiency group compared to the control group (−5.03 (−6.27, −3.72) vs. −5.81 (−7.67, −5.02) *p* = 0.040 for OCT4) and (−7.47 (−8.78, −6.27) vs. −8.5 (−10.75, −7.14), *p* = 0.035 for NANOG. Differences in the DAZL gene were not significantly different (5.94 (4.82, 7.14) vs. 6.98 (5.87, 7.43) *p* = 0.097). Pearson correlation analysis indicated the existence of a moderate correlation of OCT-4 and Nanog with cervical length. Considering this information, the enhanced activity of stemness biomarkers among pregnant women diagnosed with cervical insufficiency may be predisposed to cervical insufficiency, and its predictive accuracy remains to be noted in larger population sizes.

## 1. Introduction

Cervical insufficiency (CI) is the inability of the uterine cervix to retain a pregnancy in the second trimester, characterized by cervical shortening and dilation in the absence of uterine contractions. Although the estimated incidence of CI in the general obstetric population is estimated to be lower than 1%, it reaches up to 8% in women with a history of previous mid-trimester pregnancy loss [1]. Several risk factors have been implicated including prior treatment for cervical dysplasia (LEEP, cold-knife conization), mechanical cervical dilation and evacuation procedures, congenital uterine anomalies, and maternal connective tissue diseases [1].

**Figure 1:**
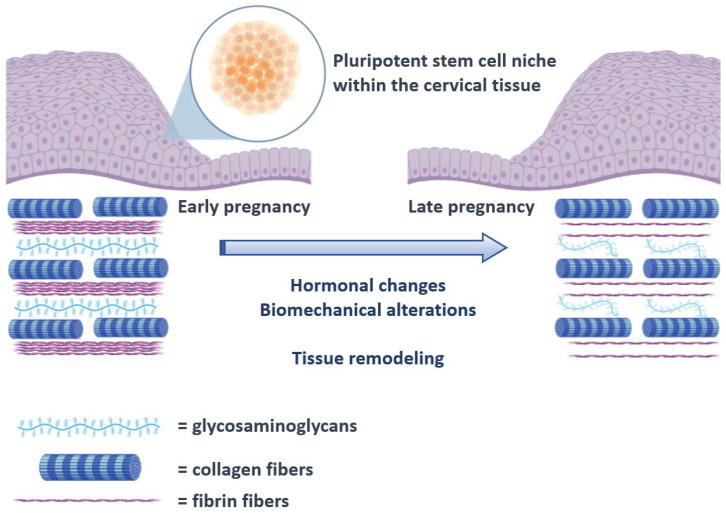
Schematic representation of the proposed process of cervical remodeling during pregnancy, under the influence of stem cells. The hormonal changes and biomechanical alterations that are observed as the pregnancy course progresses are thought to exert a proinflammatory state that progressively triggers cervical tissue remodeling that will ultimately result in cervical ripening. The process is already known to involve several cytokines including interleukins and the tissue necrosis factor a (TNF-a) [4,5]. In the present schematic representation, activation of the pluripotent cervical stem cell niche is proposed to interact with these factors and direct tissue remodeling.

The human cervix consists of fibrous connective tissue, predominantly of collagen and glycosaminoglycans. The cervical collagen network is composed of collagen type I and III and accounts for approximately 70% of the tissue’s dry weight, while muscle tissue accounts for only about 10–15%. Although the pathophysiology of CI remains unknown, it is well established that remodeling of the connective tissue is important for cervical ripening. Previous studies comparing the histological changes of the cervix in pregnant and non-pregnant women have shown that collagen concentration decreases up to 50% in the third trimester of pregnancy, while its solubility increases up to 80–90% [2]. This disorganization of the collagen network is essential for the softening of the cervix during pregnancy (Figure 1). Electron microscopy reveals a progressive reduction in cross-linked collagen, an increase in collagen fibril diameter along with increased space between fibrils from early to late gestation [3]. These changes are maximized during ripening leading to a loss of tissue integrity and eventually dilation of the cervix. Sudden premature activation of this pathway can promote preterm cervical ripening, followed by preterm delivery.

At the onset of labor, there is an increase in proinflammatory cytokines such as interleukin IL-1β, IL-6, IL-8, and TNF-α. An increase in these cytokines has been detected in cervicovaginal secretions in women with CI. Warren et al. discovered that the G13 allele in the IL-10 sequence, a cytokine that regulates the inflammatory response, appears more frequently in CI patients [6].

Stem cells may be an intermediate factor that connects the proinflammatory environment and the histological alterations that accompany cervical insufficiency as stem cells seem to respond to inflammatory conditions in several other tissues [7,8]. Previous researchers have observed that stem cells are directly involved in tissue remodeling through a process that is thought to be regulated by the involvement of the extracellular matrix and specifically the macromolecules that it contains [9,10]. Among these macromolecules, integrins and growth factors have been already described as mediators of the stem cell niche through an extensive crosstalk reaction with several receptors [7]. Experimental studies involving tissue engineering with extracellular matrix-mimicking biomaterials suggest that the induction of a proinflammatory state results in stem cell activation that triggers tissue proliferation and reconstruction through a process that involves the production of bioactive fragments that are termed matrikines [8,11,12]. To date, the actual mechanisms that involve tissue remodeling in cases presenting with cervical insufficiency during pregnancy remain poorly understood. The expression of stem cell markers has been previously described in the uterine cervix [9,10]; however, to date, potential differences among women with CI and healthy pregnant women have not been explored. Given the fact that cervical biopsies are accompanied by an increased risk of bleeding, and the fact that cervical swabs cannot retrieve an optimal number of stem cells, if at all present, we chose to evaluate the expression of stemness markers which was expected to be more significant and potentially more important as a potential screening tool in the clinical setting. Based on previous studies that suggest the expression of Octamer-binding transcription factor 4 (OCT-4), Deleted in Azoospermia (DAZL), and Nanog Homeobox (Nanog) stem cell markers in cervical tissue [13], we chose to evaluate their expression among women with CI and healthy pregnant women with normal cervical length. 

## 2. Materials and Methods

### 2.1. Patients, Ethical Approval, and Cervical Samples

This study was approved by the Ethical Review Board of Alexandra General Hospital. All patients who participated in the study by providing cytological samples provided written informed consent before the procedure. A cervical smear was obtained between 20 and 24 weeks of gestation from 41 women with a cervical length of less than 25 mm (study group) and 17 healthy women with a normal cervical length (control group). Initially, an ultrasound was performed to document the fetal heart rate, measure the length of the cervix, and estimate the fetal weight. Furthermore, obstetric history data such as gestational age, vaginal progesterone use, previous history of cervical insufficiency or preterm labor, and previous cervical surgery or trauma were recorded for each pregnant woman. Sampling was performed following the same procedure used for liquid-based cytology. Samples were stored at −80 °C for further study.

Concurrent with the retrieval of the cervical smear, a vaginal culture was also taken as this is part of the clinical routine in all patients with cervical insufficiency. To ensure that the presence of vaginal infection would not have an impact on the outcomes of cervical swabs, we retrieved cultures in control women as well and opted to include only pregnant women with normal vaginal flora.

### 2.2. RNA Extraction, Reverse Transcription, and Real-Time PCR

RNA was extracted using a commercially available kit (Monarch Total RNA Miniprep Kit, New England Biolabs Inc., Ipswich, MA, USA), which enables high-throughput purification of total RNA from up to 96 cultured-cell samples using silica-membrane RNeasy 96 plates. The RNA extracted from cervical cells was used to obtain complementary DNA (cDNA) with reverse transcription, using LunaScript RT SuperMix Kit (New England Biolabs). Quantitative Real-time PCR was performed with Light Cycler 480II (Roche Molecular Biochemicals, Mannheim, Germany) using the SYBR Luna Universal qPCR Master Mix kit (New England Biolabs Inc.). The solution for OCT4, DAZL, Nanog, and G6PD as control gene consisted of 10 μL Master mix, 1 μL of Forward Primer (10 pm/μL), 1 μL of Reverse Primer (10 pm/μL), 3 μL H_2_O. The primers used for this trial were provided by Eurofins Genomics. The primers and the hybridization probes used for this trial were provided by TIB-MOLBIOL (Table 1). The qRT-PCR reaction cycling profile was 30 s at 95 °C, 1 cycle, 5 s at 95 °C, and 30 s at 60 °C, 40 cycles. The 2^−ΔΔCT^ method was used to calculate the relative transcript abundance. All qRT-PCR reactions were repeated twice.

### 2.3. Statistical Analysis

Statistical analysis was performed using the SPSS 20.0 program (IBM Corp. Released 2011. IBM SPSS Statistics for Windows, Version 20.0. IBM Corp., Armonk, NY, USA). The Delta-Delta Ct method was used to evaluate differences among the two groups using the formula 2^−ΔΔCT^, where ΔΔCt = ΔCt (in the cervical insufficiency group) – ΔCt (in the control group). Evaluation of the normality of distributions was performed with graphical methods and the Kolmogorov–Smirnov analysis. Univariate logistic regression analysis (Enter method) was carried out in order to predict the possibility of cervical insufficiency using the following factors: gravidity, history of preterm birth or cervical insufficiency, history of prior cervical conization, age, body mass index, and differential expression of OCT-4, Nanog, and DAZL.

A predictive nomogram was developed using the results of the logistic regression analysis in Python v.3.10.8 using the Orange 3.33 data mining software. Evaluation of the predictive performance of classification and regression trees, probabilistic analysis using naïve Bayes classification, and random forest analysis was also performed to compare the predictive accuracy of the logistic regression analysis to that of more sophisticated methods. A receiver operative curve diagram was developed to permit graphical representation of our findings. 

## 3. Results

Overall, 58 pregnant women were included in the present study. Of those, 41 were diagnosed with cervical insufficiency, whereas 17 healthy women with no history of prior preterm birth or procedures in their cervix were included as the control group. The baseline differences of both groups are presented in Table 2. No other significant differences were observed, including differences in their body mass index, age, or parity. 

### 3.1. G6PD Expression

G6PD is an essential enzyme in the pentose phosphate pathway assuring normal oxidizing processes in the red blood cells. This enzyme is encoded by a human X-linked gene, which is always present in the genetic material of all cells. Thus, this gene was used as a control gene in the current study, indicating by its presence the existence of genetic material in the samples under study. G6PD expression was noted in all women, indicating that we were able to evaluate all of them for the expression of OCT-4, Nanog, and DAZL.

### 3.2. OCT-4, Nanog and DAZL Expression

OCT-4 and Nanog were expressed in all women, indicating the potential importance of these genes in cervical samples. DAZL expression, on the other hand, was more problematic as we were able to identify its expression in 15 cases with cervical insufficiency (36.6%) and 8 controls (47), therefore, rendering potentially difficult the actual screening of women with index. 

**Figure 2:**
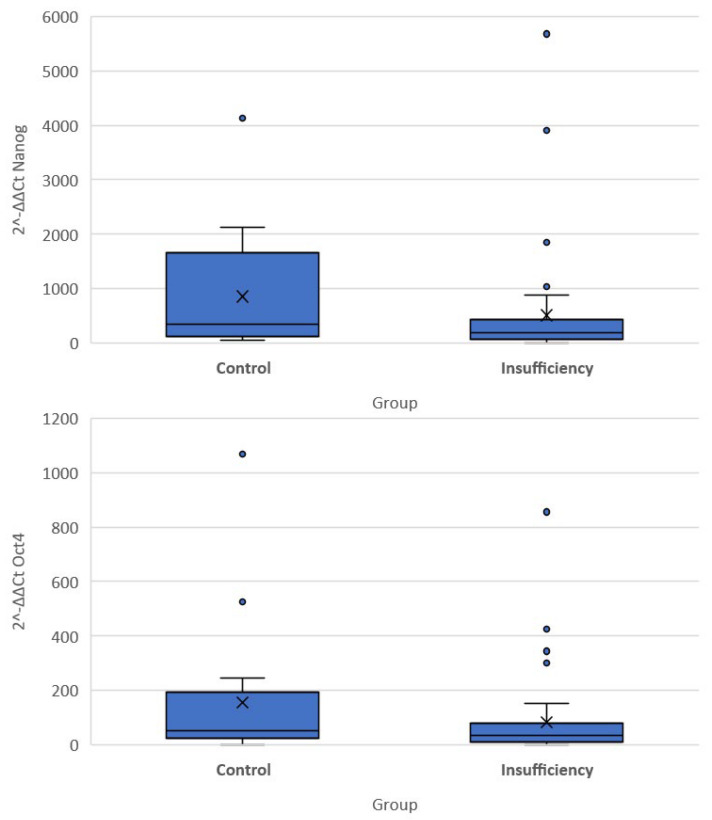
Box plots of 2^−ΔΔCT^ values of the Nanog and Oct-4 genes.

The quantitative expression of stemness markers per case included in the present cohort is presented in Table 3. Significant differences were noted in the differential expression (compared to the G6PD activity) of all three genes among the two groups of women. Of note, the expression of OCT-4 and NANOG was higher in the cervical insufficiency group compared to the control group (−5.03 (−6.27, −3.72) vs. −5.81 (−7.67, −5.02) *p* = 0.040 for OCT4 and −7.47 (−8.78, −6.27) vs. −8.5 (−10.75, −7.14), *p* = 0.035 for NANOG). Differences in the DAZL gene were not significantly different (5.94 (4.82, 7.14) vs. 6.98 (5.87, 7.43) *p* = 0.097). There was no significant difference in expression of DAZL as measured by qRT-PCR (*p* = 0.463), whereas the result was very close to statistical significance for OCT-4 (*p* = 0.082) and significant for NANOG (*p* = 0.046) (Figure 2).

**Figure 3:**
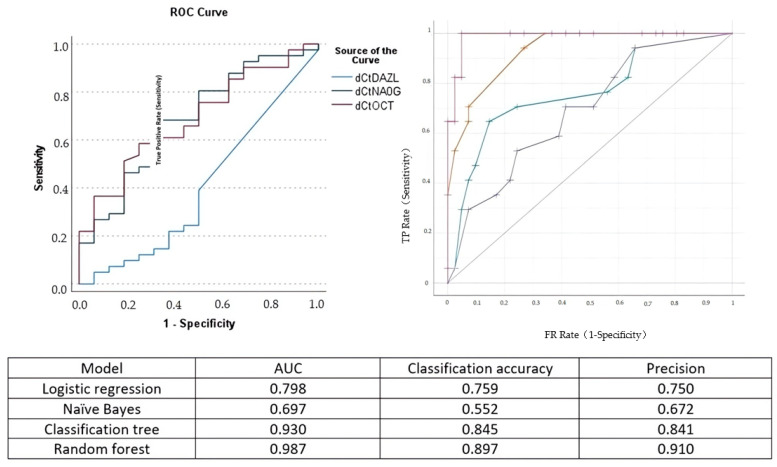
Left side: receiver operating characteristics (ROC) curve for the prediction of cervical insufficiency of DAZL (blue line), Nanog (green line), and OCT-4 (purple line). Right side: ROC analysis of the performance of logistic regression (green curve), naïve Bayes (blue line), classification and regression tree (yellow line), and random forest (purple line) analysis. The model area under the curve (AUC), classification accuracy, and precision are presented in the accompanying Table.

The Pearson correlation analysis indicated that the correlation of OCT-4 and Nanog expression with cervical length was moderate (*Spearman rho =* −*0.387* and *rho =* −*0.402*, respectively). The correlation of DAZL with cervical length was poor (*Spearman rho = 0.033*). The area under the curve for the detection of cervical insufficiency was 0.687 for OCT-4, 0.686 for Nanog, and 0.401 for DAZL, respectively (Figure 3). 

Comparing logistic regression analysis to naïve Bayes, classification and regression trees, and random forest analysis, we observed that its predictive accuracy in determining cervical insufficiency was increased compared to naïve Bayes analysis but inferior to that of random forest analysis (Figure 3).

## 4. Discussion

The findings of our study suggest the presence of increased activity of stemness markers in cervical swab specimens of pregnant women diagnosed with cervical insufficiency as their expression is significantly higher compared to that of specimens from healthy control pregnant women. Moreover, there seems to be a direct correlation between the expression of OCT-4 and Nanog and the actual cervical length, and even though the predictive accuracy of the model was moderate, the use of random forest analysis permitted optimization of the predictive accuracy of the model.

Previous studies have demonstrated the presence of stem cells in the uterine cervix [3,14]. Specifically, cancer stem cells’ existence was first described in SiHA and HeLa cell lines [15]. The modification in the expression of specific transcription factors can affect stem cell differentiation and promote tumorigenesis in women with cervical cancer. OCT4 overexpression and loss of SOX2 expression are strongly associated with a poor prognosis in patients with cervical cancer, an effect that may be attributed to structural changes of the extracellular matrix of these tissues that becomes more submissive to the invasion of cancer cells [16]. In 2012, Ting-Ting Gu et al. have shown that cytoplasmic expression of the Nanog gene in mesenchymal stem cells in cervical cancer is an indicator of disease progression [17]. However, no studies have been conducted to evaluate the association between stemness markers’ expression and cervical remodeling in women with cervical insufficiency. 

OCT4, which is encoded by POU5F1, belongs to the POU (Pit-Oct-Unc) transcriptional factor family and plays an essential role in maintaining pluripotency and self-renewal ability of embryonic stem cells [18]. The OCT4 gene can generate three mRNA isoforms, OCT4A, OCT4B, and OCT4B1. Among these, only the OCT4A, which has an 87% identical amino acid sequence to the mouse OCT4, can retain stem cell pluripotency. OCT4 has been shown to be the principal regulator of cell reprogramming and plasticity. Its overexpression can generate hematopoietic and neural progenitors and in combination with small molecules has proven to be sufficient to reprogram fibroblasts into induced pluripotent stem cells [19,20,21]. In early embryo development, reducing its expression by half leads to the differentiation of pluripotent stem cells into trophoblasts, while less than twice the overexpression triggers the differentiation of embryonic stem cells into primary endoderm and mesoderm cells [22]. 

DAZL is a protein located in the cytoplasm and nucleus of fetal germ cells and in the cytoplasm of developing oocytes. Both its RNA and protein are not found in the ovaries of postmenopausal women, while its transcripts are significantly low in men with spermatogenesis disorders [23]. In DAZL knock-out mice, there is a remarkable loss of late embryonic germ cells and complete disruption of gametogenesis, demonstrating the role of DAZL in the proliferation and differentiation of germ cells [24]. Furthermore, DAZL gene expression decreases as embryonic stem cells differentiate, while it is maintained in germline but not in somatic tissue, except gonads [25]. Thus, the fact that its expression is related only to the multipotent stage makes it a suitable marker for pluripotent/multipotent cells. 

The Nanog gene plays a critical role in determining the cell fate of intracellular mass during embryonic development, retaining the pluripotent epiblast, and preventing differentiation into primary endoderm [26]. Nanog knock-out mice fail to develop beyond the blastocyst stage due to the absence of the epiblast [26]. High levels of Nanog are crucial for the self-renewal of embryonic stem cells, while its expression is reduced during differentiation. 

Stem cells are present in most adult human tissues, and they appear to be able to differentiate in various cell lines under the influence of specific factors. Over the last few years, stem cell transplantation therapy has shown promising results in promoting wound healing. Zhang et al. have shown that human-induced pluripotent stem cell-derived mesenchymal stem cells can facilitate cutaneous wound healing by promoting collagen synthesis and neoangiogenesis [27]. Furthermore, Kubosch et al. have demonstrated that synovium-derived stem cells possess great chondrogenic potential, thus they can be used in cartilage repair [28].

The uterine cervix is a fibromuscular structure which primarily aims to reduce the possibility of ascending infection to the uterus and fallopian tubes as well as to support the pregnancy course by diminishing the possibility of preterm birth. Pathology reports suggest that the histological composition of the cervix changes throughout the life course of women as collagen stiffness increases linearly with age, whereas parity is directly associated with a reduction in its percentage [2]. The mechanical properties of the cervix have been described during the past decade and seem to be extremely crucial in terms of supporting pregnancy and reducing the possibility of preterm birth [29]. Specifically, it seems that while collagen maintains a stable percentage of the overall tissue, it is completely restructured under the influence of hormones and biomechanical alterations that normally occur as the uterus expands (Figure 2) [30]. Experimental studies have already proposed various alternative models of tissue remodeling that may support tissue remodeling, including constant evolution and reorganization of the elastic fiber network as well as proteoglycan and matricellular protein defects [31,32]. Concerning the elastic fiber network, clinical studies have shown that the dispersion of the fibers during pregnancy is more heterogeneous compared to non-pregnant women [33]. 

It should be noted that the structural changes that occur during pregnancy are not universally seen in the entire cervix. Novel studies that use quantitative 3D second-harmonic generation microscopy have suggested that the reorientation of collagen fibers is predominantly seen in the cervical canal in the inner cervical zone [34]. It is important to mention that experimental studies also suggest that the remodeling process of preterm birth as a result of an inflammatory environment differs compared to the remodeling process that normally occurs at term pregnancy [35].

The impact of stem cells on the extracellular matrix and vice versa seems to be extremely important [36]. These cells reside within tissues in the stem cell niche which is supported by various cell types as well as soluble factors [37]. The interplay of these cells with the extracellular matrix directly affects the composition of the latter part, thus determining its mechanical properties [38]. As it was previously stated, this process is mediated by bioactive fragments that are termed matrikines [39,40]. Some of those fragments protect stem cells from cytokines that induce apoptosis [41]. Inflammatory pathways seem to mediate extracellular matrix remodeling with neutrophils and M1 macrophages secreting a milieu of inflammatory cytokines, including interleukins, tumor necrosis factor a (TNF-a), and interferon gamma which are considered early factors that trigger the cascade of inflammation [42,43]. This triggers a cascade of production of several other inflammatory proteins including matrix metalloproteinases, neutrophil elastase, heparinase, and cathepsin which help modulate the extracellular matrix environment [8]. Under the influence of all these factors, the extracellular matrix undergoes a process of remodeling which results in transition from a rigid to a softer form [44,45]. Gradually, a vicious circle is being estabished as the “softer” extracellular matrix enhances inflammatory activation of mesenchymal stromal cells to induce monocyte production and relocation [46]. Taking this information into consideration, one could assume that the onset of an inflammatory/proinflammatory activity in the human cervix could result in activation of local stem cells through this “softening” of the extracellular matrix as previous studies have shown that the expression of pluripotent cells is increased in environments that support soft and low-contractility cells [47]. Experimental studies have already suggested that the proliferation of mesenchymal stem cells with the use of biological factors such as tropoelastin may promote tissue regeneration [48]. 

To summarize, the induction of a proinflammatory environment may trigger the expression of several cytokines that modulate the extracellular matrix and its elastic properties. This in turn is able to trigger the expression of stem cells which seem to be mechanosensitive as they seem to respond to the actual mechanical properties of the extracellular matrix [49,50]. The activation of stem cells further enhances the proinflammatory stimuli through the expression of monocytes and this in turn helps further expand extracellular matrix remodeling. As several extracellular components degrade during this process, the produced bioactive fragments (matrikines) protect the stem cells from apoptosis, thus inducing the maintenance of the whole process. 

### Study Limitations

Our study is mainly limited by the relatively small number of included patients which renders potentially suboptimal the power of our study. However, we believe that our findings should be regarded as preliminary and may serve as a pilot for future studies, as to date, the proposed association between stemness markers, stem cells, and cervical insufficiency has not been proposed by other researchers. Another potential limitation relies in the assumption of the expression of stemness markers by stem cells that may be more prevalent in cervices with insufficiency. In our study we did not investigate the presence of stem cells; therefore, it remains unknown whether the expression of stemness markers may accurately reflect the histological pattern of the cervix of these women. In the clinical setting, however, it is easier to reproduce our findings as the expression of stem cells is not considered safe with the use of cervical swab, and a histological expression is not considered safe as it is accompanied by heavy bleeding during pregnancy. 

## 5. Conclusions and Implications

The findings of our study propose an enhanced activity of stemness biomarkers among pregnant women diagnosed with cervical insufficiency. It remains unknown, however, if the differential expression may trigger the onset of preterm birth or if it is a secondary effect that is triggered in the presence of mechanical, proinflammatory, and hormonal alterations that occur during the pregnancy course. Therefore, further studies are needed to evaluate its diagnostic accuracy in samples retrieved during the first trimester of pregnancy, and these should ideally focus on patients at increased risk of developing cervical insufficiency (history of prior preterm birth, second trimester abortion, cervical conization, etc.). The investigation of its prognostic accuracy in patients diagnosed with cervical insufficiency is also of particular importance, as it remains unknown whether these women would benefit from an early surgical intervention with cervical cerclage along with the use of vaginal progesterone before they develop progressive cervical shortening.

## Figures and Tables

**Table 1:** Oligonucleotides used during Real Time PCR.

	Deleted in Azoospermia-Like (DAZL) Gene
**Primers**	***Sequence***
DAZL S DAZL A	gCTATgTTgTACCTCCggTTA gCCCgACTTCTTCTAAAgATg
**Probes**	***Sequence***
DAZL FL	TTTCAgAgggTggAgTAgCTTCATg-FL
DAZL LC	ACTgAACATTCATTTggACAACTTCAgCT
	**OCT-4**
**Primers**	***Sequence***
OCT-4 S	AAgCAgAAACCCTCgTg
OCT-4 A	ACTCggACCACATCCCT
**Probes**	***Sequence***
OCT-4 FL	AACAAATTCTCCAggTTgCCTC-FL
OCT-4 LC	CACTCggTTCTCgATACTggTTCgC
	**Nanog**
**Primers**	***Sequence***
Nanog S	AgATgCCTCACACggAgACT
Nanog A	CATCTgCTggAggCTgAggTA
**Probes**	***Sequence***
OCT-4 FL	AACAAATTCTCCAggTTgCCTC-FL
OCT-4 LC	CACTCggTTCTCgATACTggTTCgC

**Table 2:** Baseline patient characteristics.

	Cervical Insufficiency	Control	*p*-Value
Maternal age	29 (15–41)	36 (23–46)	0.001
Gestational age at sampling	23.1 (20.9–23.7)	22.7 (16.7–23.5)	0.760
Primiparity	0/17	4/41	0.310
History of cervical insufficiency	4/41	0/17	0.310
History of preterm birth	2/17	4/41	0.819
Cervical conization	1/17	5/41	0.660

**Table 3:** Expression of G6PD, OCT, Nanog and DAZL in the selected cohort.

Cases	G6PD	OCT	NANOG	DAZL	dCtOCT	dCtNANOG	dCtDAZL
T1	27.53	26.77	24.73	0	−0.76	−2.8	0
Τ2	29.09	22.45	20.8	0	−6.64	−8.29	0
Τ3	28.03	29.89	28.31	0	1.86	0.28	0
Τ4	30.05	25.08	23.07	0	−4.97	−6.98	0
Τ5	28.3	32.29	30.76	0	3.99	2.46	0
Τ6	26.14	20.8	22.65	0	−5.34	−3.49	0
Τ7	30.18	35	32.1	0	4.82	1.92	0
Τ8	27.45	23.92	20.45	0	−3.53	−7	0
Τ9	31.77	27.19	23.96	0	−3.98	−7.21	0
Τ10	29.56	25.8	22.9	0	−3.76	−6.66	0
Τ11	27.46	22.29	19.74	0	−5.17	−7.72	0
Τ12	28.14	21.73	19.02	0	−6.41	−9.12	0
Τ13	25.91	19.33	16.69	0	−6.58	−9.22	0
Τ14	26.74	19.5	17.15	0	−7.24	−9.59	0
Τ15	29.83	23.97	21.52	0	−5.86	−8.31	0
Τ16	29.06	20.33	17.13	0	−8.73	−11.93	0
Τ17	27.84	21.93	19.04	0	−5.91	−8.8	0
Τ18	29.17	24.73	22.19	34	−4.44	−6.98	4.83
Τ19	28.24	24.47	23.19	34.52	−3.77	−5.05	6.28
Τ20	27.71	20.49	17.69	35	−7.22	−10.02	7.29
Τ21	29.27	25.59	22.93	26.95	−3.68	−6.34	−2.32
Τ22	27.31	22.28	19.84	35	−5.03	−7.47	7.69
Τ23	28.68	22.09	18.89	0	−6.59	−9.79	0
Τ24	29.43	25.53	22.54	35	−3.9	−6.89	5.57
Τ25	27.67	24.76	21.94	35	−2.91	−5.73	7.33
Τ26	28.33	22.54	19.63	0	−5.79	−8.7	0
Τ27	28.84	25.06	20.88	33.66	−3.78	−7.96	4.82
Τ28	29.84	23.8	21.65	0	−6.04	−8.19	0
Τ29	28.03	23.51	19.41	35	−4.52	−8.62	6.97
Τ30	28.32	19.89	17.47	30.32	−8.43	−10.85	2.04
Τ31	28.91	22.77	20.95	0	−6.14	−7.96	0
Τ32	28.28	23.96	21.45	35	−4.32	−6.83	6.72
Τ33	28.4	23.47	20.46	35	−4.93	−7.94	6.6
Τ34	30.08	24.36	21.24	35	−5.72	−8.84	4.92
Τ35	30.1	20.36	17.63	35	−9.74	−12.47	4.9
Τ36	30.46	28.78	25.86	34.28	−1.68	−4.6	3.82
Τ37	26.2	21.01	19.73	34.28	−5.19	−6.47	8.08
Τ38	29.57	24.32	22.58	0	−5.25	−6.99	0
Τ39	27.89	24.67	21.69	0	−3.22	−6.2	0
Τ40	30.88	22.64	22.11	0	−8.24	−8.77	0
Τ41	29.06	27.52	23.17	35	−1.54	−5.89	5.94
C1	29.4	21.9	18.78	35	−7.5	−10.62	5.6
C2	28.12	22.31	19.68	35	−5.81	−8.44	6.88
C3	26.98	22.84	20.61	0	−4.14	−6.37	0
C4	27.16	21.01	18.66	35.4	−6.15	−8.5	8.24
C5	28.45	23.14	21.21	0	−5.31	−7.24	0
C6	27.55	21.86	19.17	0	−5.69	−8.38	0
C7	26.6	18.76	15.71	34.08	−7.84	−10.89	7.48
C8	29.2	19.14	17.19	0	−10.06	−12.01	0
C9	28.32	23.15	18.56	35	−5.17	−9.76	6.68
C10	29.45	22.54	20.56	35	−6.91	−8.89	5.55
C11	26.32	22.78	19.77	0	−3.54	−6.55	0
C12	27.7	19.75	16.78	35	−7.95	−10.92	7.3
C13	29.6	20.56	18.54	0	−9.04	−11.06	0
C14	28.72	23.18	19.65	0	−5.54	−9.07	0
C15	26.98	22.56	21.35	0	−4.14	−5.63	0
C16	27.6	21.34	20.57	34.68	−6.26	−7.03	7.08
C17	26.75	22.89	18.89	0	−4.86	−7.86	0

## Data Availability

Data will be available upon reasonable request.
